# BRAF^**V600E**^ Metastatic Synovial Sarcoma Treated with BRAF & MEK Inhibitors Achieves Complete Response. A Case Report & Literature Review

**DOI:** 10.32604/or.2026.070233

**Published:** 2026-03-23

**Authors:** Daniel Burg, Aryeh Babkoff, Omer Or, Noam Olshinka, Jonathan Abraham Demma, Mohamad Adila, Marc Wygoda, Philip Blumenfeld, Judith Diment, Masha Galiner, Yusef Azraq, Daniela Katz, Petachia Reissman, Sadie Ostrowicki, Gabriella Sebbag, Narmine Elkhateeb, Anat Hershko Moshe, Dania Jaber, Adi Hollander, Limor Rubin, Aviad Zick

**Affiliations:** 1 Faculty of Medicine, The Hebrew University of Jerusalem, Jerusalem, Israel; 2 Orthopedic Department, Hadassah Medical Center and Faculty of Medicine, Hebrew University of Jerusalem, Jerusalem, Israel; 3 Surgical Department, Hadassah Medical Center and Faculty of Medicine, Hebrew University of Jerusalem, Jerusalem, Israel; 4 Radiotherapy Institute, Sharett Institute for Oncology, Hadassah Medical Center and Faculty of Medicine, Hebrew University of Jerusalem, Jerusalem, Israel; 5 Pathology Department, Hadassah Medical Center and Faculty of Medicine, Hebrew University of Jerusalem, Jerusalem, Israel; 6 Radiology Department, Hadassah Medical Center and Faculty of Medicine, Hebrew University of Jerusalem, Jerusalem, Israel; 7 Helmsley Cancer Center, Shaare Zedek Medical Center, Jerusalem, Israel; 8 Department of Surgery, The Hebrew University School of Medicine, Shaare Zedek Medical Center, Jerusalem, Israel; 9 Department of Biology, University of Texas, Austin, TX, USA; 10 Department of Molecular Biology & Biochemistry, Rutgers University, Piscataway, NJ, USA; 11 Department of Medicine, Allergy and Clinical Immunology Unit, Hadassah Medical Center, Jerusalem, Israel; 12 Oncology Department, Sharett Institute for Oncology, Hadassah Medical Center & Faculty of Medicine, Hebrew University of Jerusalem, Jerusalem, Israel

**Keywords:** Synovial sarcoma, B-Raf proto-oncogene, serine/threonine kinase^V600E^, serine/threonine kinase inhibitor, mitogen-activated protein kinase kinase inhibitor, case report

## Abstract

**Background:**

—Synovial sarcoma is a rare soft tissue sarcoma. Treatment of synovial sarcoma includes surgery, radiation, pazopanib, and chemotherapy. Targeted therapies, such as B-Raf proto-oncogene, serine/threonine kinase (BRAF) inhibitors, are emerging as a potential treatment option. We describe the sixth case of a BRAF^V600E^ synovial sarcoma, the first extra-thoracic case. This case is the first to show a complete pathological response to BRAF & mitogen-activated protein kinase kinase (MEK) inhibitors.

**Case description:**

—We treated a 22-year-old male with a left groin BRAF^V600E^ synovial sarcoma with doxorubicin, Ifosphamide & Sodium 2-Mercaptoethanesulfonate. When we identified BRAF^V600E^ in the tumor, the BRAF^V600E^ and MEK inhibitors (dabrafenib & trametinib) were initiated, followed by surgery, with a complete pathological response. Nine months after the surgery, a local recurrence prompted the resumption of dabrafenib & trametinib followed by radiotherapy, resulting in complete radiological response and the development of hemophagocytic lymphohistiocytosis treated with corticosteroids with resolution of symptoms.

**Conclusion:**

—Panel sequencing of synovial sarcoma can identify targetable mutations. Treatment of BRAF^V600E^ synovial sarcoma with dabrafenib & trametinib can lead to complete pathological response and prolonged radiological response, as well as the rare adverse event of hemophagocytic lymphohistiocytosis. Prospective clinical trials are needed to evaluate the efficacy and safety of BRAF^V600E^ & MEK inhibitors as a therapeutic approach in BRAF^V600E^ synovial sarcoma.

## 1 Introduction

Synovial sarcoma is a soft tissue sarcoma that represents approximately 5%–10% of soft tissue sarcomas [1]. The cell of origin of synovial sarcoma is unknown, but is thought to be the myogenic progenitor cells [2]. Synovial sarcoma usually occurs in younger adults, at a mean age of 35 years. The tumor can develop in different organs, such as the lung [3], but it is more prevalent in the lower extremities. The tumor usually presents as a slow-growing mass, with a mean duration from symptoms to diagnosis of approximately two years [4]. Synovial sarcoma usually presents as a non-specific heterogeneous mass [1]. Histologically, it is composed of monomorphic spindle cells and an epithelioid component with variable differentiation [1,3]. Genetically, a pathognomonic t(X:18) translocation characterizes more than 95% of synovial sarcoma. The t(X:18) translocation results in a *SS18*:*SSX* fusion [5].

The overall 5-year survival rate of people with synovial sarcoma ranges from 59% to 75%. The 10-year cancer-specific survival rates are: 69% for localized disease, 43% for regional disease, and 8.9% for metastatic disease. The estimated 5-year and 10-year cancer-specific survival rates when stratified by age groups are 83% and 75% for children/adolescents, and 62% and 52% for adult [6]. The treatment options for synovial sarcoma include surgery, radiotherapy, and systemic treatments, such as chemotherapy and pazopanib [7], along with T-cell-based therapies targeting NY-ESO-1 [8]. Surgery is the treatment of choice in localized disease and, in many cases, it is preceded or followed by radiotherapy. Radiotherapy improves patients’ 5-year overall survival rate by 8.4 ± 1.0% [9]. Chemotherapy for local disease may be beneficial in treating high-risk primary extremity synovial sarcoma. In patients treated with ifosphamide, as a single agent or in combination with other agents, the disease-specific survival rate at 2 years was 96%, compared to 87% in those who did not receive chemotherapy. After 4 years, the disease-specific survival was 67% in the untreated group, in comparison to 88% in the ifosphamide group [10]. In metastatic disease, cytotoxic chemotherapy achieves a response rate of 27% in synovial sarcoma, contrasted with the 16%–18% response rate observed across soft tissue sarcomas. While chemotherapy remains the established cornerstone of treatment, it does not cure the disease [11,12]. Another treatment option is the use of Pazopanib. A retrospective study from a single institute showed a clinical benefit (stable disease or partial response) in 46% of patients with advanced or metastatic soft tissue sarcoma treated with pazopanib [13].

Synovial sarcoma exhibits a range of structural variations and mutations within its molecular landscape. In more than 95% of cases, the *SS18*-*SSX* translocation is observed (*SSX1* is present in two-thirds of synovial sarcoma cases, *SSX2* in one-third, and *SSX4* is rarely observed) [14]. The most common point mutations occurred in the genes *SETD2* (4%), *ABL1* (4%), *SESN2* (3%), *EPAS1* (3%), *SLX4* (3%), *DROSHA* (3%), and *SHOC2* (3%) [15].

The BRAF^V600E^ mutation is reported in several cases of synovial sarcoma. *BRAF* is a proto-oncogene that is a component of the Mitogen-activated protein kinase (MAPK) pathway. This pathway is involved in different cellular activities, such as proliferation [16]. An active BRAF mutation results in uncontrolled cellular growth and tumorigenesis and occurs in many types of malignancies, including melanoma and colon cancer [17,18]. Over 90% of these mutations are a missense mutation in exon 15 that results in a replacement of Valine in position 600 to Glutamic acid (V600E) [17,18]. Throughout the years, patients have been treated with several small molecules that inhibit BRAF^V600E^ and its downstream effectors [17,18]. Patients diagnosed with unresectable or metastatic BRAF^V600E/K^ melanoma, treated with BRAF and MEK inhibitors, have a 5-year progression-free survival of 19% and overall survival of 34% [17,18]. Furthermore, the use of dabrafenib & trametinib has shown promising results in various types of cancers, including anaplastic thyroid and biliary tract cancer [19,20]. The ROAR study is a phase 2 basket trial investigating the effectiveness and safety of dabrafenib & trametinib in patients with BRAF^V600E^-mutated rare cancers. The ROAR study demonstrated promising efficacy and safety profiles of the combination therapy with diverse response rates, durations of response, and survival outcomes in different patient cohorts. Across different rare cancer types, the combination therapy resulted in varying median overall survival periods, ranging from 13.5 months to an indefinite period. Disease progression was found to be the main cause of death in all patient groups, with no deaths attributed to the experimental medication. The treatment was well tolerated, and its efficacy was observed regardless of tumor type, suggesting its potential as a valuable therapeutic approach for patients with rare BRAF^V600E^ cancers. Following the study results, the FDA expedited approval of dabrafenib in combination with trametinib for patients ages six and above with unresectable or metastatic BRAF^V600E^ solid tumors. This approval applies to patients who have experienced disease progression after prior treatment and have limited alternative treatment options, excluding colon cancer [21].

Whether or not BRAF inhibition benefits patients with BRAF^V600E^ synovial sarcoma is not established. We identified five reported cases of BRAF^V600E^ synovial sarcoma. The first two cases were part of a basket trial testing the efficacy of vemurafenib in BRAF^V600E^ non-melanoma tumors [22], a male participant below the age of 47 and a female participant aged between 48 and 59. The presentation, location, and detailed clinical course were not reported. The overall survival was 3.7 and 2 months for the male and the female patients, respectively. Another study tested BRAF^V600E^ in 69 patients with synovial sarcoma [23]. Out of 67 patients, 2 were found with BRAF^V600E^. The first case was a 32-year-old woman who presented with chest pain due to a 12 cm mass. Biopsy showed spindle cell monophasic synovial sarcoma morphology with *SS18*:*SSX2* fusion. The patient was treated with doxorubicin & ifosfamide followed by tumor resection. This treatment resulted in complete remission for 18 months, followed by pulmonary metastasis. A biopsy from the pulmonary mass showed a BRAF^V600E^ mutation. On immunohistochemistry (IHC), the tumor was positive for BRAF^V600E^ and phosphorylated extracellular signal-regulated kinase (pERK) staining. After disease recurrence, the patient was treated with several chemotherapy lines, including trabectedin. The patient did not receive any BRAF^V600E^ targeted therapy due to the lack of ongoing trials at the time. Overall, the patient lived 43 months from initial disease presentation to death. The second case is a 23-year-old woman who presented with Horner’s syndrome due to a 4.3 cm mass in the superior mediastinum. Due to hemothorax secondary to the tumor, immediate resection of the tumor was performed. Histologically, the tumor showed spindle cell monophasic synovial sarcoma morphology with *SS18*:*SSX2* fusion. On IHC, the tumor was positive for BRAF^V600E^ and pERK staining. Five months after resection, the patient presented with right arm and shoulder pain due to recurrence. The patient was treated with several treatment modalities, including radiation, chemotherapy and pazopanib. Eventually, she was treated with dabrafenib & trametinib. Under this treatment, the patient was in remission for 7.5 months, followed by a local recurrence. A biopsy from the recurrent tumor showed BRAF^V600E^ and pERK staining. Next-generation sequencing revealed an NRAS^Q61K^ mutation, which was absent from the primary tumor [23].

Lastly, a 15-year-old male with an intrathoracic synovial sarcoma. Histologically, the tumor showed spindle cell monophasic synovial sarcoma morphology with *SS18:SSX1* fusion. The patient was treated with resection of the tumor followed by adjuvant chemo-radiotherapy. The tumor recurred, and the patient was treated with several lines, including pazopanib and chemotherapy. Panel sequencing showed a BRAF^V600E^ mutation, and the patient received vemurafenib. Four months after the initiation of vemurafenib, a partial response was seen. The patient is still alive with an overall survival of 19 months and a progression-free survival of 6 months [24].

Here we report about a 22-year-old male patient who presented with a left groin BRAF^V600E^ synovial sarcoma with complete response to BRAF & MEK inhibitors. As far as we know, this is the sixth reported case of synovial sarcoma harboring a BRAF^V600E^ mutation and is the first reported extra-thoracic synovial sarcoma harboring a BRAF^V600E^ mutation treated with BRAF & MEK inhibitors with a complete radiological response.

This study was approved by the ethics committee of the Hadassah Medical Center, with the reference number: HMO-0346-12. Handwritten informed consent was obtained from the patient. Besides, this study was prepared according to the CARE case report guideline, and a CARE checklist [25] is provided. Please see Supplementary Material S1 for more details.

## 2 Case Report

**Patient Information**—A 22-year-old patient, treated in the Hebrew University-Hadassah Medical Center, Jerusalem, Israel, presented with a four-month history of lower back pain, a two-month history of a growing mass in his left lower abdomen, and a month of anorexia and weight loss (5 kg in a month). The patient had no past medical history and did not take any medication. Family history included a grandmother with sarcoma and a grandmother with vaginal cancer.

**Clinical Findings**—On physical examination, a 20 cm left lower quadrant abdominal mass was palpated with left leg weakness.

**Figure 1:**
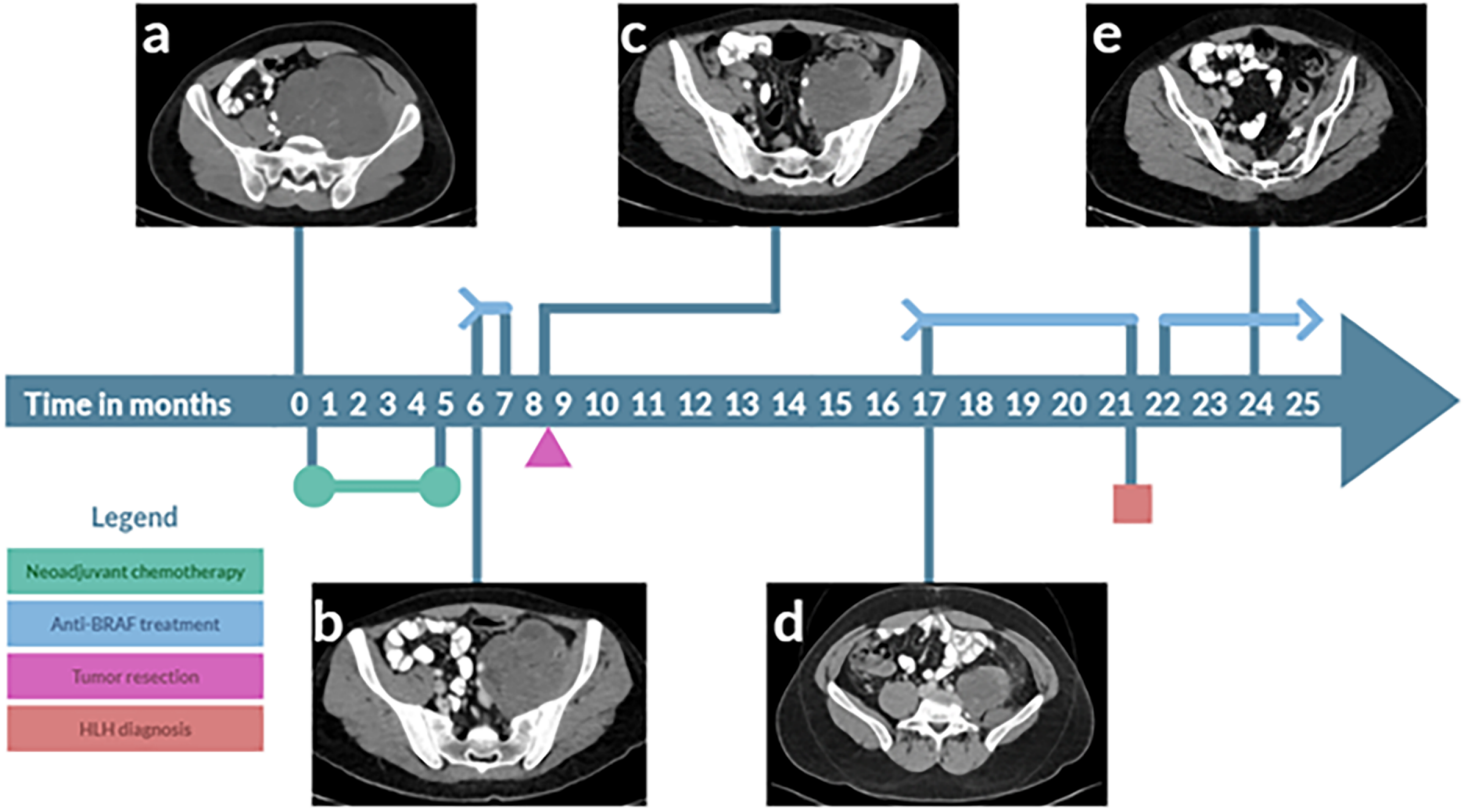
Treatment of BRAF^V600E^ synovial sarcoma with dabrafenib & trametinib leads to a prolonged complete response. The timeline illustrates the initiation of neoadjuvant chemotherapy, followed by a switch to treatment with dabrafenib & trametinib (blue arrow). Computerized tomography (CT) images were taken at various points to assess response. During treatment, hemophagocytic lymphohistiocytosis (HLH) occurred (red box). The arrowhead indicates an ongoing response. A CT scan with intravenous contrast transverse images evaluated by a board-certified radiologist (**a**) shows a large, heterogeneously enhancing mass in the left psoas muscle, measuring 13 × 13 × 15 cm. The mass contains internal vascularity and multiple areas of necrosis, extending from the L3 vertebra down to the left hip joint. It crosses the midline to the right, displacing the iliac vessels, left ureter, and pelvic structures to the right side. (**b**) Reduction in mass size observed after 6 months of doxorubicin & ifosphamide treatment, followed by (**c**) further reduction in tumor size 2 months later, following treatment with dabrafenib & trametinib. (**d**) Nine months after surgery, there was a new mass at the surgical bed, involving the descending colon. (**e**) After seven months of dabrafenib & trametinib treatment, there is no evidence of disease.

**Diagnostic Assessment—**A left-groin abdominal ultrasound demonstrated a solid, heterogeneous, slightly vascular mass. A pelvic Computed Tomography (CT) scan evaluated by a board-certified radiologist showed a large heterogeneously enhancing mass infiltrating the left Psoas muscle, consistent with sarcoma (Fig. 1a).

**Figure 2:**
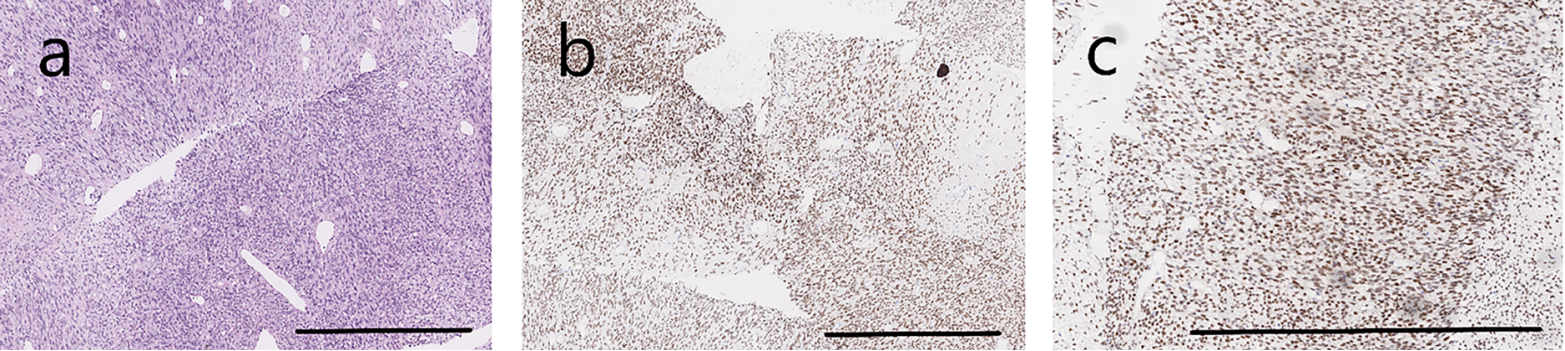
Monophasic synovial sarcoma harbors a BRAF^V600E^ mutation. (**a**) Synovial sarcoma, monophasic, spindle cell type at 10× magnification. (**b**) Immunohistochemistry stains were positive for SS18-SSX at 10× magnification and (**c**) TLE1 at 20× magnification. Scale bars: 200 µm in (**a,b**); 100 µm in (**c**).

A biopsy from the pelvic mass shows cores of a cellular spindle cell neoplasm, showing crowded spindle cells with indistinct clear cytoplasm and small to medium sized oval to elongated nuclei. The stroma is collagenous with few myxoid foci and the cells have a vague fascicular arrangement. Numerous mitoses are present (Fig. 2a). The tumor was positive for BCL6 corepressor (BCOR) and the SS18 subunit of BAF chromatin remodeling complex (SS18)–SSX fusion (SS18 fused to an SSX family member—SSX1/SSX2/SSX4) (Fig. 2b), with focal, faint positivity for CD99 (MIC2 cell-surface glycoprotein) and S-100 protein. Desmin, actin, Mucin 4 (MUC4), and pan-cytokeratin (pan-CK) were negative. The findings are suggestive of synovial sarcoma, monophasic spindle-cell type. A SYT translocation was identified using fluorescence *in situ* hybridization (FISH) and BRAF^V600E^ mutation using Oncomine^™^Comprehensive Assay Plus.

**Therapeutic Intervention—**The case was discussed in a multidisciplinary tumor board, which recommended neoadjuvant chemotherapy. Before starting chemotherapy, the patient underwent sperm preservation using electroejaculation. The patient initiated a course of Adriamycin, Ifosphamide and Sodium 2-Mercaptoethanesulfonate (MESNA), a regimen known as AIM. Following the first cycle of AIM, the patient was admitted to the urgent care department due to neutropenia fever. A CT scan revealed a collection of fluid and air near the anorectal area, while the pelvic mass displayed no significant change in size. These findings were consistent with a fistula or perforation likely resulting from electroejaculation. The patient received antibiotics and Granulocyte colony-stimulating factor(G-CSF), resulting in a significant improvement in his condition. Following this improvement, chemotherapy was resumed, and a subsequent CT scan after two cycles indicated stable disease. After two additional cycles of AIM, an CT scan showed a reduction in tumor size (Fig. 1b). After six cycles of AIM, the patient was admitted to the urgent care department due to a high fever and headaches. Blood tests showed elevated troponin and a later workup was positive for COVID-19. Therefore, due to a targetable mutation, dabrafenib & trametinib treatment was initiated.

**Follow-up and Outcomes**—After 19 days, a CT scan revealed an additional reduction in tumor dimensions (Fig. 1c). The patient was treated with open resection of the tumor involving the psoas, left iliac vein, and left internal iliac artery. During surgery, several lumbar and sacral nerve roots were resected, with a root nerve damage affecting L2-L5. Due to massive bleeding during surgery, the patient needed 12 units of blood and 9 units of fresh frozen plasma. The post-operative process was normal. Post-surgery pathology showed a complete response; the patient did not receive any adjuvant treatment.

After nine months, a CT scan showed a new space occupying lesions at the surgical bed (Fig. 1d). Treatment with dabrafenib & trametinib was re-initiated, and the patient was referred to a multidisciplinary team, which recommended continuing dabrafenib & trametinib alongside radiotherapy. Radiotherapy was given three months after the re-initiation of dabrafenib & trametinib. Five months after the re-initiation of dabrafenib & trametinib, the patient developed symptoms of immune dysregulation, such as weakness, low-grade fever, and pancytopenia. These, along with splenomegaly and elevated inflammatory markers (C-Reactive Protein 30 mg/dL), led to suspicion of secondary Hemophagocytic Lymphohistiocytosis (HLH). Further investigations confirmed the diagnosis with elevated ferritin (6700 ng/dL), triglycerides (275 mg/dL), and soluble IL-2 receptor (16,790 U/mL). According to the diagnostic criteria for HLH used in the HLH-2004 trial the patient fulfilled five positive criteria out of nine [26]: splenomegaly, cytopenia’s, hypertriglyceridemia (fasting, >265 mg/dL) hyperferritinemia and elevated sIL-2 receptor. His H-Score was 170, corresponding with 40%–54% probability of HLH, making the diagnosis of HLH probable, investigation for secondary HLH was done. Viral etiologies were ruled out include Epstein-Barr virus (EBV IgM and EBV PCR were negative, EBV VCA IgG and EBV EBNA IgG were positive that suggest previous infection), Cytomegalovirus (CMV IgM, CMV IgG, CMV PCR were negative), HIV, Sars-Cov-2 and other respiratory viruses. There were no clinical signs of autoimmune disease and laboratory studies were negative for APLA, ANA and ANCA. Adult-onset Still’s disease was less probable due to the absence of rash, neutrophilia, pharyngitis or arthritis. There was no previously known immunodeficiency. His bone marrow biopsy did not shown any signs of malignancy or lymphoma. There were numerous small granulomas. B-raf and SS18-SSX immunostains were negative. His IgG levels were low 582 mg/dL, IgM levels were 17 mg/dL and IgA levels were 104 mg/dL.

Dabrafenib & trametinib were temporarily halted, and high-dose corticosteroids initiated. The patient’s condition improved with reduced ferritin and LDH levels and resolution of cytopenia. Two weeks after symptom resolution, treatment with Dabrafenib with Trametinib was resumed with prednisone. Initially at dabrafenib 150 mg once daily & trametinib 0.5 mg one daily and after one week with dabrafenib 150 mg twice daily & trametinib 2 mg once daily. Imaging tests, including CT and MRI scans, indicated no evidence of disease (Fig. 1e). After 22 months of treatment, the patient is currently free of disease and continues treatment with dabrafenib with trametinib and 2.5 mg prednisone.

## 3 Discussion

Metastatic synovial sarcoma is characterized with poor prognosis, with limited treatment options including cytotoxic chemotherapies or pazopanib. We present a case of a 22-year-old male diagnosed with BRAF^V600E^ synovial sarcoma treated with dabrafenib & trametinib, with a complete pathological response. On disease recurrence dabrafenib & trametinib treatment was reinitiated with complete radiological response. During dabrafenib & trametinib treatment HLH occurred and was treated with corticosteroids.

HLH is a life-threatening multisystem syndrome characterized by overstimulation of the immune system, leading to hyperinflammation and multi-organ damage [26,27]. Estimated overall mortality rates in adults are 40%–50% [28,29]. Several methods were suggested to aid in the diagnosis of HLH, including the HLH-2004 criteria and HScore [26,30,31]. HLH can be either primary or secondary. Primary HLH typically presents in infancy and is characterized by genetic mutations that impede cytotoxic function of natural killer (NK) and cytotoxic T cells. Conversely, secondary HLH presents in adults and is associated with a predisposing condition causing immune dysregulation [27], which can include infections, malignancy, autoimmune diseases, or drugs [12,27,32,33]. Several cases of HLH due to anti-BRAF/anti-MEK combination therapy were reported, mostly, in patients diagnosed with metastatic melanoma [34−36]. No formal guidelines were suggested regarding the management of HLH induced by anti-BRAF/anti-MEK therapy. Nonetheless, in the reported cases, management was based on discontinuation of the medications, supportive care and occasionally, initiation of corticosteroids [34−36]. The reintroduction of anti-BRAF/anti-MEK therapy has shown no recurrence in most patients and was considered safe [34−36].

NY-ESO-1, a cancer-testis antigen expressed in approximately 80% of synovial sarcomas [37,38]. In this process, T-cells are harvested from the patient and genetically engineered to express a T-cell receptor (TCR) that specifically recognizes the NY-ESO-1 antigen. The patient undergoes lymphodepletion to reduce the number of existing T-cells. The engineered T-cells are infused back into the patient along with interleukin-2 (IL-2) to support their growth and activity. These modified T-cells specifically target and destroy NY-ESO-1 expressing cancer cells, resulting in tumor shrinkage and improved survival rates [37]. In patients with synovial sarcoma treated with NY-ESO-1 TCR, an objective response was observed in 14/18 patients. The three-year survival rate was 38%, and the 5-year survival rate was 14%. The median overall survival is 24.3 months [38].

In the case of this patient, several questions emerge. Although *BRAF* mutations in synovial sarcoma are very rare, treatment is effective. Does this suggest a role of the RAS-RAF-MEK-ERK pathway in synovial sarcoma?

The patient has BRAF^V600E^, NY-ESO-1 positive sarcoma. What should be the treatment sequence of chemotherapy, targeted therapy and T-cell therapy? Would longer neo-adjuvant or adjuvant dabrafenib & trametinib treatment prevent the appearance of metastatic disease? For how long should dabrafenib & trametinib treatment be continued in the light of a complete radiological response?

To our knowledge, this is the first documented case of a patient with synovial sarcoma developing HLH due to dabrafenib & trametinib treatment. This case highlights the importance of considering HLH as a rare but serious complication of targeted therapy, especially in patients receiving BRAF and MEK inhibitors. The case raises the question if the complete radiological response and development of HLH are unrelated or did the drug treatment trigger an immune response leading to both? Besides, what is the most favorable way to manage HLH due to anti-BRAF/anti-MEK therapy?

Continued basic research and clinical trials will help to further establish the role of the RAS-RAF-MEK-ERK pathway in synovial sarcoma, as well as the efficacy and safety of dabrafenib & trametinib treatment in BRAF^V600E^ synovial sarcoma.

**Strengths and limitations**—The strength of this case is a clear diagnosis (monophasic synovial sarcoma with *SS18:*:*SSX* fusion & BRAF^V600E^); a response to dabrafenib & trametinib both initially with a complete pathological response and following disease recurrence with a complete radiological response and a follow-up of ~22 months. The limitations of this work include the non-generalizability of a single case and a correlation of HLH to BRAF & MEK therapy.

## 4 Conclusion

This case report adds to the limited body of published evidence concerning targeted therapy in BRAF^V600E^ synovial sarcoma, providing insight into both the notable response to BRAF inhibitor & MEK therapy and the side effects associated with BRAF/MEK inhibitors. Our report highlights the clinical utility of BRAF & MEK-targeted therapy in synovial sarcoma and underscores the importance of genomic profiling in guiding treatment decisions for this rare malignancy. This case sheds light on the potential risk of developing secondary HLH as a serious, life-threatening complication associated with anti-BRAF/anti-MEK therapy. While BRAF/MEK inhibitors offer a promising treatment strategy, clinicians should be vigilant about rare but critical immune dysregulation side effects, such as HLH, particularly in patients undergoing long-term therapy. Prospective studies are essential to further define the role of genome-matched therapy in managing this phenotypically and genetically complex disease.

**Patient Perspective:** The patient was happy to stop chemotherapy and start a biological pill. The fever & fatigue can become worse. He enjoys his ability to take vacations including sailing and is looking towards continuing his life and getting married.

## Supplementary Materials



## Data Availability

The authors confirm that the data supporting the findings of this study are available within the article.

## Supplementary Materials

The supplementary material is available online at https://www.techscience.com/doi/10.32604/or.2026.070233/s1.
