# Supplementary material for: BRAFV600E Metastatic Synovial Sarcoma Treated with BRAF & MEK Inhibitors Achieves Complete Response. A Case Report & Literature Review
Source: Oncol Res. 2026 Mar 23;34(4):38. doi: 10.32604/or.2026.070233 (PMC13040339; doi:10.32604/or.2026.070233)
Supplement: Supplementary file 1 [file OncolRes-34-70233-s001.pdf]

CARE Checklist of information to include when writing a case report

Topic	Item No	Checklist item description	Reported on Page Number/Line Number	Reported on Section/Paragraph
Title	1	The diagnosis or intervention of primary focus followed by the words "case report"	page 1 line 1	Title page
Key Words	2	2 to 5 key words that identify diagnoses or interventions in this case report, including "case report"	page 2 line 20-23	Abstract page
Abstract (Structured summary)	3a	Background: state what is known and unknown; why the case report is unique and what it adds to existing literature.	page 2 line 2-7	Abstract page
	3b	Case Description: describe the patient's demographic details, main symptoms, history, important clinical findings, the main diagnosis, interventions, outcomes and follow-ups.	page 2 line 8-14	Abstract page
	3c	Conclusions: summarize the main take-away lesson, clinical impact and potential implications.	page 2 line 15-19	Abstract page
Introduction	4	One or two paragraphs summarizing why this case is unique **(may include references)**	page 3-4 line 1-40	Introduction
Patient Information	5a	De-identified patient specific information	page 5 line 1	Patient Information
	5b	Primary concerns and symptoms of the patient	page 5 line 2-4	Patient Information
	5c	Medical, family, and psycho-social history including relevant genetic information	page 5 line 4-6	Patient Information
	5d	Relevant past interventions with outcomes	page 5 line 4	Patient Information
Clinical Findings	6	Describe significant physical examination (PE) and important clinical findings	page 5 line 7-8	Clinical Findings
Timeline	7	Historical and current information from this episode of care organized as a timeline	page 5 line 13-26	Figure 1
Diagnostic Assessment	8a	Diagnostic testing (such as PE, laboratory testing, imaging, surveys).	page 5-6 line 9-7	Diagnostic Assessment
	8b	Diagnostic challenges (such as access to testing, financial, or cultural)	page 5-6 line 9-7	Diagnostic Assessment
	8c	Diagnosis (including other diagnoses considered)	page 6 line 5-6	Diagnostic Assessment
	8d	Prognosis (such as staging in oncology) where applicable	page 5-6 line 9-7	Diagnostic Assessment
Therapeutic Intervention	9a	Types of therapeutic intervention (such as pharmacologic, surgical, preventive, self-care)	page 6-7 line 12-16	Therapeutic Intervention
	9b	Administration of therapeutic intervention (such as dosage, strength, duration)	page 6-7 line 12-16	Therapeutic Intervention
	9c	Changes in therapeutic intervention (with rationale)	page 6-7 line 12-16	Therapeutic Intervention

Follow-up and Outcomes	10a	Clinician and patient-assessed outcomes (if available)	page 6-7 line 12-16	Follow-up and Outcomes
	10b	Important follow-up diagnostic and other test results	page 6-7 line 12-16	Follow-up and Outcomes
	10c	Intervention adherence and tolerability (How was this assessed?)	page 6-7 line 12-16	Follow-up and Outcomes
	10d	Adverse and unanticipated events	page 6-7 line 12-16	Follow-up and Outcomes
Discussion	11a	A scientific discussion of the strengths AND limitations associated with this case report	page 7-8 line 18-30	Discussion
	11b	Discussion of the relevant medical literature **with references**	page 7-8 line 18-30	Discussion
	11c	The scientific rationale for any conclusions (including assessment of possible causes)	page 7-8 line 18-30	Discussion
	11d	The primary "take-away" lessons of this case report (without references) in a one paragraph conclusion	page 7-8 line 18-30	Discussion
Patient Perspective	12	The patient should share their perspective in one to two paragraphs on the treatment(s) they received	page 8 line 30-33	Patient Perspective
Informed Consent	13	Did the patient give informed consent? Please provide if requested	**Yes** ☑	**No** ☐

*As the checklist was provided upon initial submission, the page number/line number reported may be changed due to copyediting and may not be referable in the published version. In this case, the section/paragraph may be used as an alternative reference.
